# Metachronous primary cancer of the tongue and malignant lymphoma of the small intestine: A case report

**DOI:** 10.1097/MD.0000000000024806

**Published:** 2021-02-19

**Authors:** Keisuke Sugimoto, Shinji Uejima, Yumiko Uchiyama, Reita Yasue, Kazuya Nambu, Jun Ishikawa, Yoshiro Koma, Takako Akita, Taketomo Toh, Takehiro Fujimoto

**Affiliations:** a Department of Oral and Maxillofacial Surgery, Iwata City Hospital, Iwata; b Department of Oral and Maxillofacial Surgery, Nagoya University Hospital; c Department of Oral and Maxillofacial Surgery, Nagoya University Graduate School of Medicine, 65 Tsurumai-cho, Showa-ku, Nagoya; d Department of Oral and Maxillofacial Surgery, Tokoname City Hospital, 3-3-3 Asukadai, Tokoname, Aichi, Japan.

**Keywords:** multiple primary cancer, small intestine malignant lymphoma, tongue cancer

## Abstract

**Rationale:**

Oral cancer often causes secondary primary cancers in the upper gastrointestinal tract. However, there are no reports of secondary primary cancers in patients with oral squamous cell carcinoma and malignant lymphoma of the small intestine. This report describes a case of metachronous multiple primary cancers of the tongue and small intestine malignant lymphoma.

**Patients concerns:**

The patient was admitted to our department with the chief complaint of pain in the right tongue. Partial tongue resection and supraomohyoid neck dissection were performed. One year after surgery, the patient experienced abdominal pain and bloody stools.

**Diagnosis:**

Diffuse large B-cell lymphoma (DLBCL) was diagnosed via histological examination.

**Interventions:**

A terminal ileum resection was performed. Postoperatively, the patient received 6 courses of rituximab, cyclophosphamide, doxorubicin hydrochloride, vincristine, and prednisone (R-CHOP).

**Outcomes:**

Five years after his initial diagnosis, there is no evidence of recurrence, metastasis, or other primary cancer.

**Lessons:**

Oral cancer patients should always be followed up owing to a possibility of malignant tumors in other areas.

## 1 Introduction

The incidence of secondary primary cancers has been increasing because treatment outcomes in patients with squamous cell carcinoma of the head and neck have improved.^[1–6]^ The incidence of secondary primary cancers in patients with oral cancer is higher than that in patients with malignant tumors at other sites.^[5–7]^ Some studies have reported that the incidence of secondary primary cancers is approximately 6% to 10% per year in patients with oral cancer.^[6–8]^ Moreover, it is suggested that secondary primary cancers often occur, especially in the upper gastrointestinal tract.^[4–7]^ Some studies reported that the incidence of secondary primary cancers in the upper gastrointestinal tract is as high as 10% per year in patients with oral cancer.^[4–8]^ However, secondary primary cancers in patients with oral cancer rarely appear in the lower gastrointestinal tract. Small intestine malignant tumors are relatively rare, with a frequency of approximately 0.06% to 2% of all gastrointestinal malignant tumors. Small intestine malignant lymphoma accounts for approximately 2% to 4% of all small intestine malignant tumors.^[9]^ To the best of our knowledge, there are no reports of secondary primary cancers of small intestine malignant lymphoma and oral squamous cell carcinoma. Here, we report a case of malignant lymphoma of the small intestine during follow-up after tongue cancer treatment.

## 2 Case report

**Figure 1:**
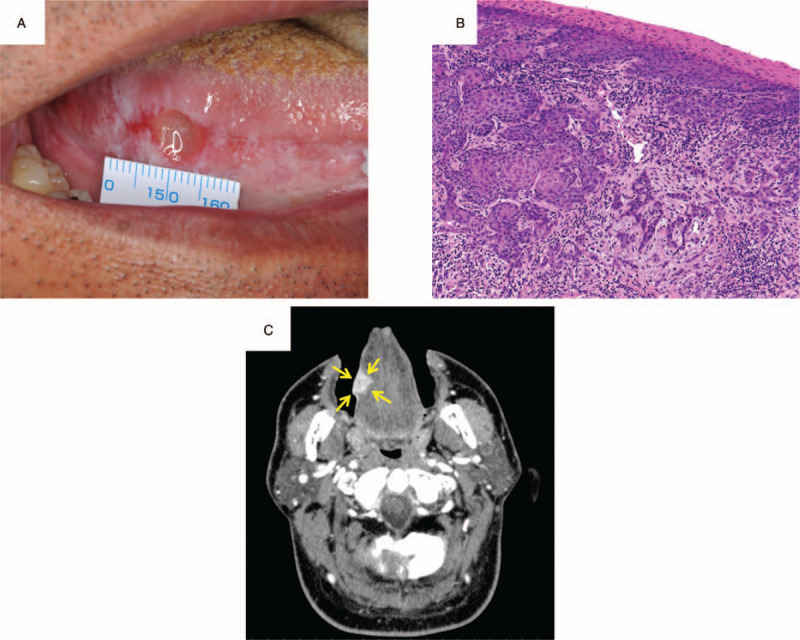
A, Photograph of the tongue configuration. B, Pathological examination of the biopsy (hematoxylin-eosin stain, original magnification ×20). C, Contrast-enhanced CT image of the axial section before treatment. CT = computed tomography.

**Figure 2:**
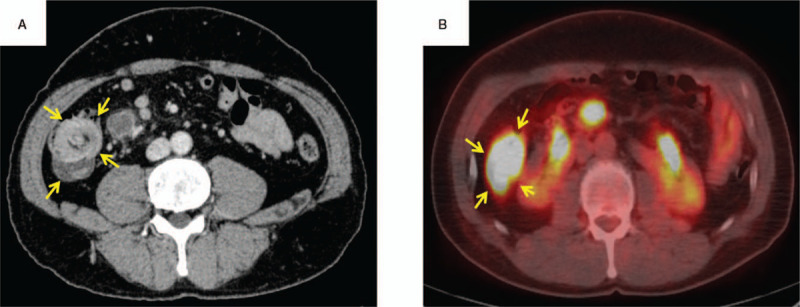
A, PET image and B, contrast-enhanced CT image of the coronal section, January 2017. CT = computed tomography; PET = positron emission tomography.

**Figure 3:**
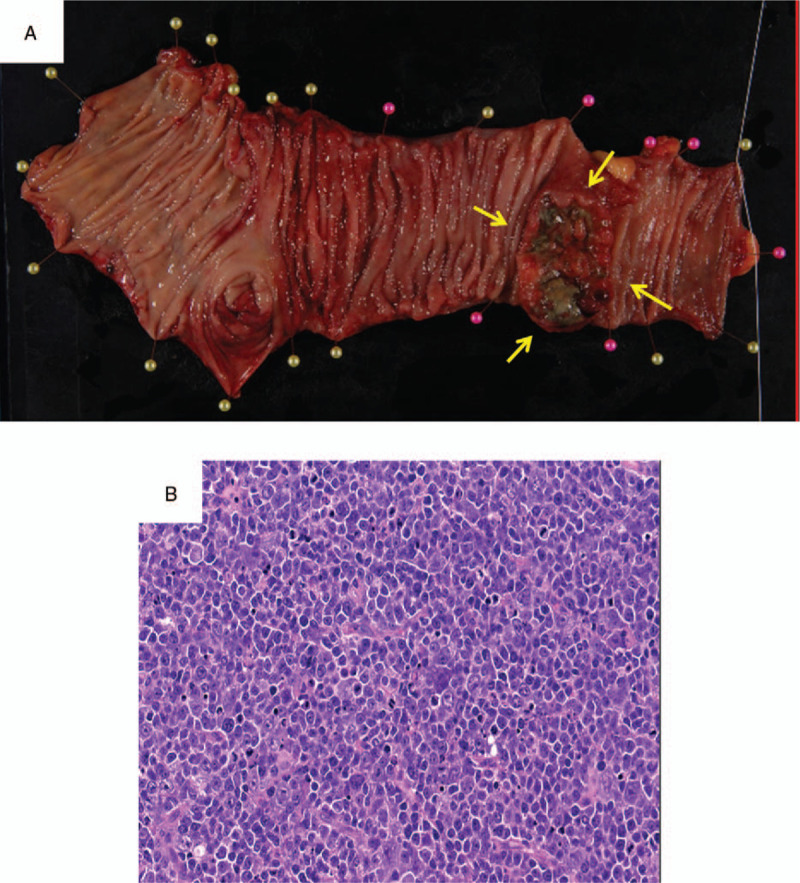
A, Photograph of the surgical specimen in the terminal ileum and B, pathological examination of resection specimen (hematoxylin-eosin stain, original magnification ×20).

**Figure 4:**
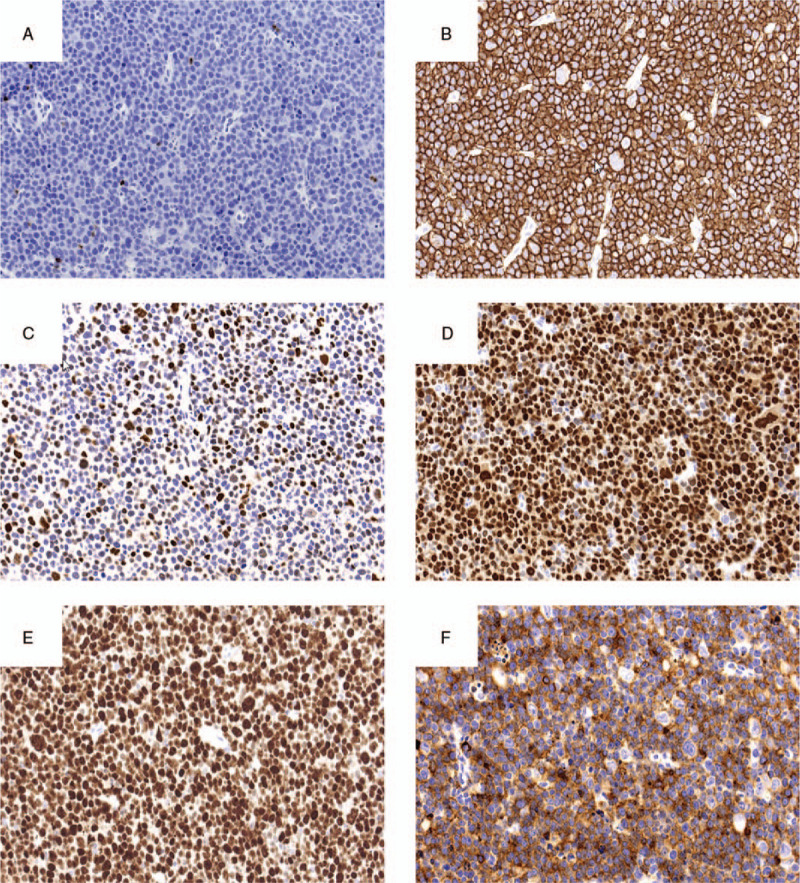
Immunohistochemical staining of A, CD3; B, CD20; C, BCL-6; D, NUM-1; E, IgM; and F, MIB-1 (original magnification ×20).

A 52-year-old man with spontaneous pain and swelling in the right tongue region consulted his regular dentist, who referred the patient to our hospital in July 2015. A lesion with an induration of 7 × 10 mm was found on the right tongue (Fig. 1A). There was an irregular surface and ulceration in the lesion. He was diagnosed with squamous cell carcinoma of the right tongue via pathological examination in August 2015 (Fig. 1B). Contrast-enhanced computed tomography (CT) showed a contrast region with a width of 18 mm and a depth of 11 mm in the longitudinal direction of the tongue (Fig. 1C). Magnetic resonance imaging (MRI) and contrast-enhanced CT showed no evidence of cervical lymph node metastasis. From the examination results, he was diagnosed with tongue cancer of the right side (cT2N0M0, at stage II). The patient underwent a partial tongue resection and supraomohyoid neck dissection in September 2015. For the resection, a safety margin of 10 mm was set from the tumor, and the depth of resection was 10 mm from the tumor throughout the circumference. The postoperative pathological diagnosis was squamous cell carcinoma, and the surgical margin was negative at all sites. After the operation, he was observed in our outpatient department. In November 2016, he had abdominal pain and bloody stool symptoms and consulted a family clinic. Since he was diagnosed with intussusception, he was referred to our gastroenterology department. Contrast-enhanced CT was performed and showed a contrast region in the terminal ileum (Fig. 2A). A lower gastrointestinal endoscopy was performed, and a tumor was found near the terminal ileum. Malignant lymphoma was suspected based on the histological examination results. Positron emission tomography (PET) showed accumulation in the terminal ileum (Fig. 2B). Terminal ileum resection was performed in department of gastroenterological surgery in March 2017 (Fig. 3A). Pathological examination of the surgical specimen revealed that the polymorphonuclear atypical cells were distributed in a sheet form (Fig. 3B). The results of immunohistochemical staining were CD3(–), CD20(+), BCL-6(+), NUM-1(+), and IgM(+) (Fig. 4A–E, respectively). The MIB-1 labeling index was 90% (Fig. 4F). Diffuse large B-cell lymphoma (DLBCL) was diagnosed via histological examination. Postoperatively, the patient received 6 courses of rituximab, cyclophosphamide, doxorubicin hydrochloride, vincristine, and prednisone (R-CHOP therapy) at department of hematology from April to August 2017. A complete hematological remission was obtained after 6 courses of R-CHOP therapy. Five years after surgery for tongue cancer, there was no evidence of recurrence in the head and neck region. In addition, no recurrent findings of the malignant lymphoma were observed as he was under regular follow-up in the hematology department.

## 3 Discussion

Secondary primary cancer refers to the occurrence of primary malignant tumors in other organs. Warren and Gate's diagnostic criteria were used to diagnose secondary primary cancer. A diagnosis of secondary primary cancer requires the following conditions: each cancer presents a definite malignant image, each is independent, and the possibility that metastasis of the other is excluded.^[1]^ In addition, those diagnosed in a period of <1 year were defined as having simultaneity, and those diagnosed in a period of ≥1 year were defined as having heterochrony.^[1–2]^ In this patient's case, it was a secondary primary cancer of tongue cancer and malignant lymphoma of the small intestine.

Malignant lymphoma was not identified on examination at the time of initial diagnosis. Since it was diagnosed >1 year later, it was diagnosed as metachronous secondary primary cancer.

In recent years, the number of secondary primary cancers has been increasing due to longevity; improvement of various diagnostic techniques; improved prognosis of the first cancer; exposure to carcinogens due to lifestyle and environmental factors, such as smoking and drinking; and genetic factors.^[6,8,10–12]^ Moreover, the relationship between workers who professionally handle pesticides such as herbicides, pesticides, and fertilizers and the occurrence of malignant lymphoma (non-Hodgkin lymphoma) has been clarified epidemiologically.^[13–16]^ In our case, the patient was a non-smoker. However, he was engaged in agriculture and was thought to be exposed to pesticides. Therefore, it was suggested that pesticide exposure might have been involved in the development of malignant lymphoma. Since the relationship between occurrence of malignant lymphoma and the use of pesticides has not been revealed clearly, further studies are needed to clarify this relationship.

After treatment of oral cancer, the incidence of secondary primary cancer is approximately 3.2%.^[6]^ To improve treatment results of secondary primary cancer, early detection and early treatment of multiple organ cancer are necessary after oral cancer treatment. Therefore, it has been pointed out that a whole-body search that regularly examines for the presence of distant metastases and secondary primary cancer is important. In oral cancer, the incidence of secondary primary cancer in the upper gastrointestinal tract is high, and upper endoscopy is useful for detecting lesions.^[6,7,17]^ However, since secondary primary cancer of the lower gastrointestinal tract, such as in our case, is rare, it is difficult to perform lower endoscopy for regular follow-ups. Therefore, PET may be useful for screening for systemic secondary primary cancer.^[18–20]^ PET is superior to other examinations, in that it can detect distant metastases and secondary primary cancer because a whole-body search can be performed in a single examination. The frequency of distant metastases varies depending on the primary site and tumor size.^[20,21]^ However, about 20% of patients with head and neck cancer have distant metastases or secondary primary cancer by PET.^[20]^ PET also contributed to the detection of lesions in our case. It was suggested that PET was one of the useful examinations for detecting lesions.

Small intestine malignancy is a relatively rare disease, with a frequency of approximately 0.06% to 3% of all gastrointestinal malignancies, and small intestine malignant lymphoma accounts for approximately 2% to 4% of all small intestinal malignant tumors.^[22,25,26]^ The age of prevalence is 55 to 60 years, but it is found in all age groups, from children to the elderly; in terms of sex, small intestine malignancies occur twice as often in men as women. The most common clinical symptoms are abdominal pain, mass palpation, weight loss, and melena. The most common histological type is DLBCL, with a frequency of approximately 30% to 63%.^[22–26]^ In our case, intussusception was first suspected based on clinical findings. Approximately 60% to 70% of adult intussusception often develops secondarily with organic abnormalities such as tumor and inflammatory intestine disease.^[27–29]^ Approximately 10% to 15% of adult intussusceptions are gastrointestinal malignant lymphomas.^[25–28]^ Since adult intussusception is often due to organic abnormalities, it was suggested that it was necessary to search for neoplastic lesions, including malignant diseases. It is often difficult to perform biopsies and histological diagnoses of gastrointestinal malignant lymphoma, and the correct diagnosis rate for intestinal malignant lymphoma with intussusception is about 27%.^[29]^ Of these correct diagnoses, 91% were diagnosed by biopsy under lower endoscopy. The incidence of small intestine malignant lymphoma intestine is 74% within 10 cm and 93% within 30 cm of the terminal ileum.^[26]^ This suggests that lymphatic tissues such as Peyer patches and lymphoid follicles develop at the terminal ileum, and malignant lymphoma is likely to occur. Therefore, most malignant lymphomas of the small intestine could be observed by colonoscopy and diagnosed by biopsy. In our case as well, a tumor was present near the terminal ileum; thus, a definitive diagnosis could be obtained by biopsy under lower endoscopy. It was suggested that the use of lower endoscopy is useful for diagnosis.

The Ann Arbor classification is generally used for staging malignant lymphoma.^[24]^ However, since malignant lymphoma of the gastrointestinal tract is mainly composed of extranodal lesions, it may deviate from the progression of the disease in the Ann Arbor classification.^[25]^ Therefore, the Lugano classification is used in addition to the Ann Arbor classification for malignant lymphoma of the gastrointestinal tract. In the Lugano classification, stage I/II1 is generally resectable and limited type, while stage II2 or higher is considered advanced stage.^[9,25,30]^ A standard treatment approach for gastrointestinal malignant lymphoma is not certain currently.

Based on the results of a comparative study of surgery and chemotherapy versus chemotherapy alone for moderate to high-grade lymphoma such as DLBCL, the surgery and chemotherapy groups had a favorable prognosis.^[9]^ Therefore, prioritizing surgery and performing postoperative chemotherapy for resectable lesions are recommended. Our case was diagnosed as stage II1 by Lugano classification. Although surgical resection was considered curative, R-CHOP therapy was combined with postoperative treatment because R-CHOP therapy is the standard treatment for DLBCL.^[9,30]^

Five years have passed since the first visit, and no evidence of new cancers, recurrence, or metastasis has been found.

## 4 Conclusion

This report suggests that oral cancer patients should always be followed up owing to a possibility of malignant tumors in other areas, and appropriate detection methods should be combined for early detection.

## Author contributions

**Conceptualization:** Keisuke Sugimoto.

**Investigation:** Shinji Uejima, Yumiko Uchiyama, Reita Yasue, Kazuya Nambu, Yoshiro Koma.

**Supervision:** Takehiro Fujimoto.

**Writing – original draft:** Keisuke Sugimoto.

**Writing – review & editing:** Keisuke Sugimoto, Jun Ishikawa, Takako Akita, Taketomo Toh, Takehiro Fujimoto.
